# Insulin secretion and action with increasing age - A comparison between Middle Eastern immigrants and native Swedes

**DOI:** 10.1016/j.heliyon.2022.e10913

**Published:** 2022-10-03

**Authors:** Nadine Fadhel Dhaher, Nael Shaat, Anton Nilsson, Louise Bennet

**Affiliations:** a Department of Medicine, Trelleborg's Hospital, Trelleborg, Sweden; b Genomics, Diabetes and Endocrinology, Department of Clinical Sciences, Lund University, Malmö, Sweden; c Department of Endocrinology, Skåne University Hospital, Malmö, Sweden; d Department of Laboratory Medicine, Lund University, Lund, Sweden; e Department of Clinical Sciences in Malmö, Lund University, Malmö, Sweden; f Clinical Research and Trial Centre, Lund University Hospital, Lund, Sweden

**Keywords:** Insulin action, Insulin secretion, Middle East, Type 2 diabetes, Iraqi-born immigrants, Native Swedes

## Abstract

**Aims:**

Little is known how insulin secretion and action change over time in populations of different ethnicities. We studied changes in insulin secretion and action with increasing age in Iraqi-born immigrants and native Swedes, and investigated if the changes were modified by region of origin.

**Methods:**

Residents of Malmö, 30–75 years of age born in Iraq or Sweden, were invited to participate in this population-based, cross-sectional study. Health examination, medical history, lifestyle, sociodemographic data, and fasting blood samples were assessed. Oral glucose tolerance tests were performed and insulin secretion (disposition index, DIo) and insulin sensitivity index (ISI) calculated using the Matsuda indices.

**Results:**

In total 1881 people participated; 1193 Iraqi- and 688 Swedish born. DIo decreased with increasing age in the total study population (β for the effect of age on ln DIo: ^-^0.018, 95% CI ^-^0.023 to ^-^0.013, *P* < 0.001), adjusted for origin, lifestyle and anthropometric measures. DIo was generally lower in Iraqis vs. Swedes (median: 12,712.9 vs. 14,659.2, *P* = 0.004), but the difference disappeared when adjusted for BMI.

Further, ISI declined with increasing age in both Iraqis and Swedes. ISI was generally lower among Iraqis compared to Swedes, (median: 76.9 vs. 102.3, *p* < .001). The difference could not be fully explained by age, sex, lifestyle, and anthropometric measures. No significant interactions were observed.

**Conclusions:**

The levels of DIo and ISI were lower among Iraqis compared to Swedes and declined with increasing age, irrespective of origin.

## 1 Introduction

Over the recent decades, immigration from the Middle East has been rapidly increasing in Northern Europe due to war and political instability in some of the Middle Eastern countries. At present, 19.6% of the Swedish population consists of residents born outside Sweden. According to a report done by Statistics Sweden in 2020, Iraqi-born citizens represent the second largest group of foreign-born persons in Sweden (N = 146 440). Malmö is the third-largest city in Sweden and hosts one of the largest Middle Eastern communities with 25.8% (2021) of its residents being born outside the country [1].

According to the National Diabetes Register (NDR) report from 2019, there are approximately 450,000 persons (>18 years) currently suffering from diabetes in Sweden with a majority of them, 98%, diagnosed with type 2 diabetes (T2D), a number that corresponds to about 4% of the adult Swedish population [2]. Compared to the native population, migrants in Europe are at higher risk of developing cardiometabolic diseases such as T2D and unhealthy lifestyles [3, 4]. Among immigrants to Sweden, a higher prevalence of well-known cardiometabolic risk factors for T2D such as obesity and hypertension have been observed [3]. Regarding immigrants from Iraq, the prevalence of T2D has been estimated to be twice as high compared to that of native Swedes [5].

The development of T2D is related to impaired insulin sensitivity and/or insulin secretion [6, 7, 8, 9]. Ethnic differences regarding insulin sensitivity and secretion have been observed in populations that have not yet developed diabetes [10, 11], thus different mechanisms are likely to contribute to diabetes development in diverse ethnic populations. For instance, in populations of Middle Eastern and Asian ethnicity, insulin deficient diabetes is more prevalent whereas in African populations insulin resistant diabetes is more common than in European populations [12, 13, 14, 15]. As for Iraqi-born individuals living in Sweden, severe insulin-deficient diabetes was shown to be almost twice as common compared to a Swedish-born control group, whereas severe insulin-resistant diabetes was less prevalent [15].

The MEDIM (impact of Migration and Ethnicity on Diabetes in Malmö), a population-based cohort including residents 30–75 years of age living in Malmö, Sweden was conducted between 2010 to 2012. MEDIM reported that the Iraqi immigrant population is at higher risk of developing T2D, display younger age at onset and have a higher prevalence of diabetes-related risk factors as compared to the native Swedish population [10]. Although MEDIM reported a higher degree of insulin resistance in the Iraqi immigrant population compared with native Swedes, insulin secretion adjusted for insulin resistance (oral disposition index, DIo) was lower among Iraqis indicating the presence of a relative insulin deficiency in the, presumably, healthy non-diabetic immigrant population [10].

Altered glucose metabolism is associated with increasing age, this includes both relative insulin resistance and beta cell dysfunction [16]. Insulin sensitivity is influenced by age and aging and impairment in insulin action is associated with a decline in responsiveness to carbohydrates, which leads to higher blood glucose levels [16]. Further, impaired insulin secretion has been shown to be more prominent in the elderly population [17, 18, 19, 20, 21]. However, the progression of insulin secretion and action over time across different populations of Middle Eastern and European ethnicities is still unknown.

The aim was to study if aging influences insulin secretion and action measured as DIo and ISI (insulin sensitivity index, eq. 1) respectively in Middle Eastern immigrants and native Swedes. In addition, we aimed to study further whether the potential effects of age were modified by country of birth. We used age as a proxy of change over time.

## 2 Methods

### 2.1 Study population

Residents of Malmö, Sweden, aged 30–75 years born in Iraq or Sweden, were invited to participate in the population-based study MEDIM, which was conducted between 2010 to 2012. The process of how the participants were recruited has previously been described in detail [10]. Briefly, the participants were randomly selected from the census register, invited by mail and phone. Individuals with severe physical or mental illness were excluded from the study. All information was provided in both Swedish and Arabic. Participants from the same socioeconomic area were invited with the Swedish-born as a control group matched for sex and age. Prior to participation, everyone signed informed consent forms, all participants underwent a health examination including anthropometrics, fasting blood samples and an oral glucose tolerance test (OGTT). Sociodemographic and lifestyle behavior data were collected via questionnaires as well as information regarding current medication and chronic diseases [10]. On the day before testing, the participants were asked not to consume tobacco or eat or drink after 10 pm. Blood samples were collected in the morning and analyzed during the study continuously. Details regarding the methods for blood sample analyses have been described earlier [22].

A flowchart describing the recruitment of MEDIM participants and response rate is shown in supp1. In total, ISI was assessed in 1212 Iraqi and 704 Swedish-born participants whereas DIo was assessed in 1193 Iraqi and 688 Swedish-born participants that were included in the analysis. The exclusion after OGTT was due to CIR (corrected insulin response, eq. 2) criteria requiring that glucose at 30 min (glc30) > 4.44 mmol/L and glc30 > f-glc [7].

### 2.2 Definitions

Insulin sensitivity index (ISI, eq. 1), insulin secretion (corrected insulin response, CIR, eq. 2) and DIo were assessed through insulin and glucose measured at 0, 30 min, 60 min and 120 min during OGTT (Matsuda indices) [7, 8, 9, 23]. The Matsuda calculation formula has been shown to have good correlation with intravenous glucose tolerance test (IVGTT), especially when used on cross-sectional data [24, 25].

Participants with diabetes confirmed by medication did not undergo an OGTT.

ISI, by Matsuda provides an estimate of hepatic and muscle insulin sensitivity, ISI corresponds inversely with insulin resistance [7, 8, 9].

(1) ISI = 10,000/√ [(f-glc (mmol/L) × f-insulin (mIE/L)) × (mean OGTT glc conc. (mmol/L) × mean OGTT insulin conc. (mIE/L))]

CIR (Corrected Insulin Response) is assessed to measure glucose-stimulated insulin secretion and provides an estimate of beta-cell function. CIR was calculated from OGTT as follows [7, 8, 9]:

(2) CIR = (100 × insulin at 30 min (mIE/L)/(glc30 (mmol/L) × (glc30–3.89 mmol/L)) and requires that glucose at 30 min (glc30) > 4.44 mmol/L and glc30 > f-glc

DIo is an estimate of beta-cell function adjusted for insulin resistance. DIo is the product of CIR and ISI [7, 8, 9].

### 2.3 Statistical analysis

Analyzes were performed using IBM SPSS Statistics 26. Data are presented in means (standard deviation, SD), numbers (percentages) or for skewed data, medians (interquartile range, IQR). All tests were two-sided and a p-value of <0.05 was considered statistically significant. Skewed variables were ln - transformed before analysis to approximate normal distributions.

The study population was divided into four age categories based on quartile cut points with approximately 540 individuals in each age category: < 39 years, 39 ≥ to <46 years, 46 ≥ to <55 years and >55 years.

Independent sample Median test was used to compare the levels of insulin secretion and insulin sensitivity across groups. Multiple linear regression models were used to explore the associations between insulin secretion, insulin sensitivity (dependent variables) and country of birth, age, sex, BMI, physical activity, and tobacco as independent variables.

### 2.4 Ethical considerations

All participants provided written informed consent and the Ethics Committee at Lund University approved the study (No. 2009/36 & 2010/561). This investigation conforms to the principles outlined in the Declaration of Helsinki [26].

## 3 Results

**Figure 1:**
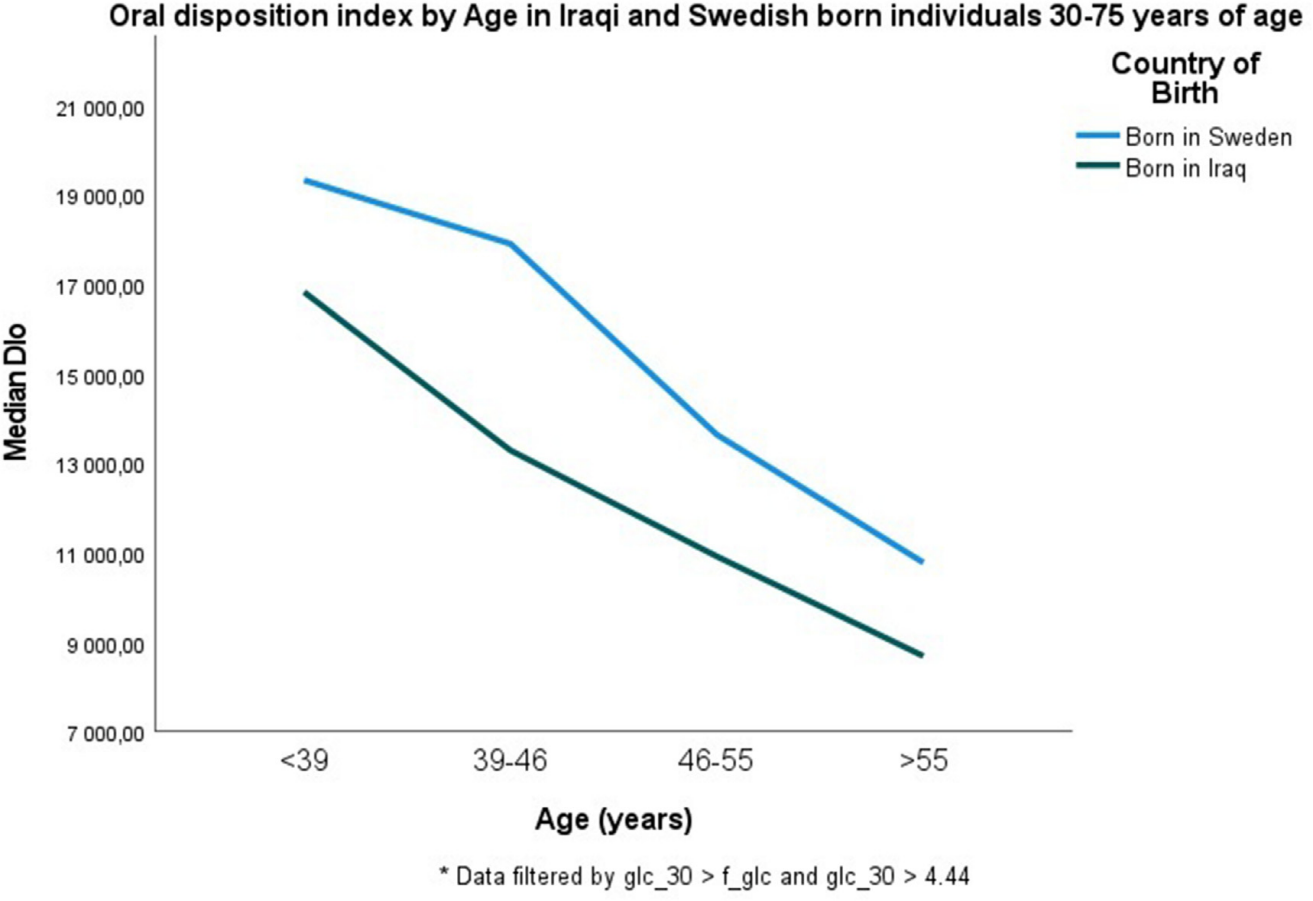

**Figure 2:**
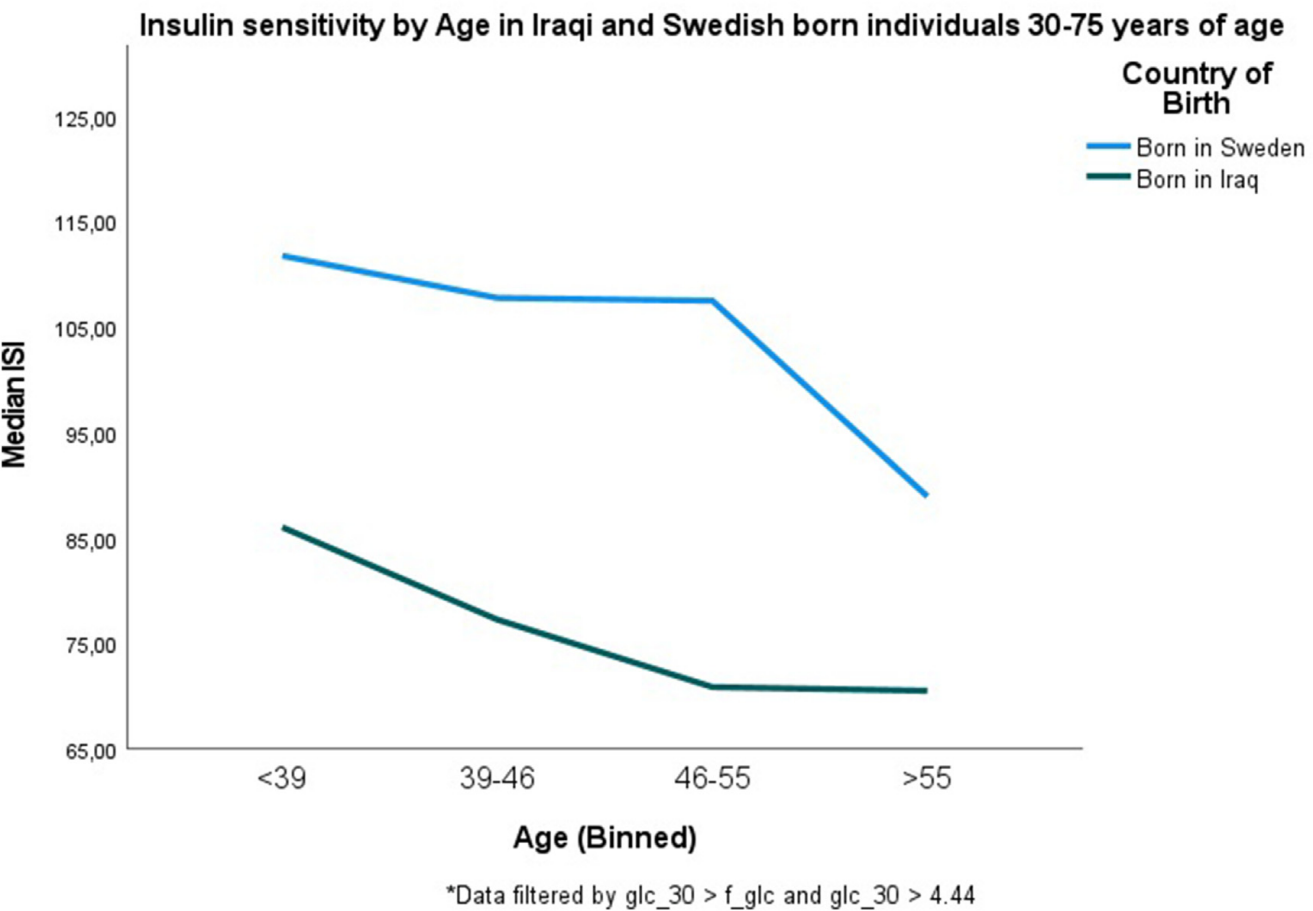

The baseline clinical characteristics of both groups are described in Table 1. Iraqi immigrants were younger, had higher BMI and were less physically active compared to the Swedish control group. Figures 1 and 2 illustrate that both insulin secretion represented by DIo, and insulin action represented by ISI decreased with age in both the Iraqi and Sweden-born groups. DIo decreased with increasing age in the total study population (β for the effect of age on ln DIo: ^-^0.018, CI ^-^0.023 to ^-^0.013, *P* < 0.001), adjusted for origin, lifestyle, and anthropometric measures. DIo was generally lower in Iraqis vs. Swedes (median: 12,712.9 vs. 14,659.2, *P* = 0.004), but the difference disappeared when adjusted for BMI. β-cell function (Figure 1) declined with increasing age following a linear pattern; however, the degree of decline in β-cell function was almost similar among the Iraqi and the Swedish population, (β ^-^0.025, *p* < .001 vs ^-^0.022, *p* < .001) per year without any adjustment and (β ^-^0.023, *p* < .001 vs ^-^0.020, *p* < .001) per year when adjusted for sex and BMI. No significant interaction was observed between country of birth and age (P_interaction_ = 0.653). Iraqi immigrants had generally lower DIo in all age categories compared to native Swedes, but the differences were not significant after adjusting for BMI (Table 2). The β coefficient was reduced by approximately 50% after adjustment for this variable, reflecting that being overweight has a great effect on insulin secretion.

**Table 1:** Characteristics of study participants Iraq and Swedish born living in Malmö.

Variable	Country of Birth	
	Iraq (N = 1193)	Sweden (N = 688)
Age (years)	45.5 (9.3)	49.3 (11.1)
Male sex, n (%)	696 (58.3)	367 (53.3)
Body mass index, kg/m^2^	29.03 (4.4)	27.1 (4.5)
Waist circumference, men (cm)	98.7 (10.6)	97.4 (11.3)
Waist circumference, women (cm)	92.3 (10.5)	88.9 (13.6)
Hours physically active/week	1.9 (2.0)	4.1 (2.4)
Total cholesterol (mmol/L)	4.9 (0.9)	5.3 (1.0)
p-LDL (mmol/L)	3.2 ​(0.8)	3.3 ​(0.9)
p-HDL (mmol/L)	1.2 ​(0.3)	1.4 ​(0.4)
p-Triglyceride (mmol/L)	1.5 ​(0.9)	1.2 ​(0.8)
Smokers, *n* (%)	285 (23.9)	175 (25.4)
Antihypertensive medication, *n* (%)	113 (9.5)	92 (13.4)
Family history of diabetes, *n* (%)	587 (49.2)	186 (27.0)
Intake of fruit or berries, less than every day, *n* (%)	1084 (90,9)	602 (87.5)
Education level ≤ HS, n (%)	856 (71.8)	559 (81.3)
ISI (mmol/L∗mIE/L−1)∗	76.9	102.3
CIR (mmol/L∗mmol/L∗mIE/L – 1)∗	169.67	147.64
DIo(mmol/L∗mmol/L∗mmol/L)∗	12,712.9	14,659.2

Crude data are presented as means (SD) or as numbers (percentages); ​family history refers to biological parents, children and/or siblings; LDL/HDL is low-density/high-density lipoprotein; HS is high school; intake of fruit or berries was self-reported and dichotomized into intake once or more a day, or less than once a day; Tobacco smoking, participants stating that they never smoked were considered none-smokers and the rest classified as smokers.

∗ Differences in medians between groups.

**Table 2:** Linear regression models with ln DIo as a dependent factor expressed as β coefficients with 95% confidence intervals.

Variable	Model 1 N = 1880 R^2^ = .004	Model 2 N = 1819 R^2^ = .068	Model 3 N = 1818 R^2^ = 0.106	Model 4 N = 1625 R^2^ = .109
Born in Sweden Born in Iraq	Reference ^-^.133∗∗ ^-^.230 to ^-^.037	Reference ^-^.146∗∗ ^-^.245 to ^-^.047	Reference ^-^.055 ^-^.154 to 0.044	Reference ^-^.031 ^-^.143 to .082
Age (years)		^-^.020∗∗∗ ^-^.024 to ^-^.015	^-^.018∗∗∗ ^-^.023 to ^-^.014	^-^.018∗∗∗ ^-^.023 to ^-^.013
Female sex Male sex		^-^.194∗∗∗ ^-^.286 to ^-^.102	^-^.207∗∗∗ ^-^.297 to ^-^.117	^-^.171∗∗∗ ^-^.266 to ^-^.076
Family history of diabetes Yes No		^-^.043∗∗∗ ^-^.061 to ^-^.025	^-^.038∗∗∗ ^-^.056 to ^-^.020	^-^.037∗∗∗ ^-^.056 to ^-^.019
Body mass index (kg/m^2^) per unit			^-^.045∗∗∗ ^-^.055 to ^-^.035	^-^.046∗∗∗ ^-^.056 to ^-^.035
Hours physically active/week				.018 ^-^.003 to .039
Current tobacco smoking (No as reference)				^-^.053 ^-^.164 to .057

P < 0.05∗, P < 0.01∗∗, P < 0.001∗∗∗.

Figure 2 illustrates ISI as a function of increasing age in the Iraqi and Swedish populations. As for insulin secretion, insulin action declined with increasing age and was significantly lower in Iraqi immigrants than native Swedes, after adjusting for covariates in the full model (Table 3). After 55 years of age, a shaper drop in ISI in the Swedish born group was observed than in the Iraqi group. There was no significant interaction between country of birth and age on ISI (*P* = 0.587). The median level of ISI among the youngest Iraqi-born immigrants (<39 years of age), was even lower than the median level of ISI among the oldest Swedish-born participants (>55 years of age) (85.98 vs 88.90) (*P* = 0.001). Across all age groups, the Iraqi immigrant population displayed significantly lower median levels of ISI compared to the Swedish population even after adjusting for sex and BMI, (<39 years of age) (85.8 vs 111.7) (*P* = 0.014), (39–46 years of age) (77.2 vs 107.7) (*P* = 0.001), (46–55 years of age) (70.8 vs 107.4) (*P* = 0.001), (>55 years of age) (70.5 vs 88.9) (*P* = 0.009).

**Table 3:** Linear regression models with ln ISI as a dependent factor expressed as β coefficients with 95% confidence intervals.

Variable	Model 1 N = 1915 R^2^ = .047	Model 2 N = 1854 R^2^ = .091	Model 3 N = 1852 R^2^ = .307	Model 4 N = 1654 R^2^ = .327
Born in Sweden Born in Iraq	Reference ^-^.288∗∗∗ ^-^.346 to ^-^.230	Reference ^-^.281∗∗∗ ^-^.342 to ^-^.221	Reference ^-^.151∗∗∗ ^-^.205 to ^-^.097	Reference ^-^.093∗∗ ^-^.154 to^−^.032
Age (years)		^-^.005∗∗ ^-^.008 to ^-^.002	^-^.003∗ ^-^.005 to .000	^-^.003∗∗ ^-^.006 to .001
Female sex Male sex (reference)		^-^.227∗∗∗ ^-^.283 to ^-^.171	^-^.245∗∗∗ ^-^.294 to ^-^.196	^-^.263∗∗∗ ^-^.315 to ^-^.211
Family history of diabetes Yes No (reference)		^-^.015∗∗ ^-^.026 to ^-^.004	^-^.008∗∗∗ ^-^.018 to .002	^-^.006 ^-^.017 to .004
Body mass index (kg/m^2^) per unit			^-^.066∗∗∗ ^-^.072 to ^-^.061	^-^.065∗∗∗ ^-^.071 to ^-^.059
Hours physically active/week				.034∗∗∗ .023–.046
Current tobacco smoking (No as reference)				.044 ^-^.017–.104

P < 0.05∗, P < 0.01∗∗, P < 0.001∗∗∗.

## 4 Discussion

### 4.1 Statement of principal findings

To the best of our knowledge, this is the first study to investigate the relationship between insulin secretion and action with respect to increasing age in a Middle Eastern immigrant population compared with a native Swedish population. The study illustrates that β-cell function, represented by DIo and insulin action, represented by ISI, both decline with increasing age in the Iraqi and Swedish-born cohorts, even after adjustment for other diabetes-related risk factors such as age, sex, BMI, family history of diabetes and health-related behaviors. Further, across all ages, the Iraqi immigrant population displayed significantly lower median levels of ISI compared to the Swedish population even after adjusting for factors such as BMI. Insulin resistance was profound in the youngest Iraqi immigrants as their ISI was similar to that in the oldest Swedish-born group.

An imbalance between insulin resistance and β-cell dysfunction contributes to the development of type 2 diabetes mellitus [6, 27, 28]. DIo is a measurement of β-cell function adjusted for insulin sensitivity, and the decline in β-cell function appears in high-risk individuals years before the development of overt diabetes [7, 9]. The β-cell has the ability to regulate insulin secretion based on changes in insulin sensitivity [29]. Our previous data showed that Iraqi immigrants have a higher risk of developing type 2 diabetes at an earlier age compared to native Swedes, which suggests that β-cell dysfunction and impaired insulin sensitivity appear at younger ages [10]. In addition, we have suggested that the impaired insulin action observed among Iraqis is not fully explained by traditional risk factors and there are ethnic differences concerning insulin action and secretion [10].

This study illustrates how DIo and ISI change over time and represents an approach to shed light on mechanisms involved in the prediabetes stage. In this study, the level of DIo in young Iraqis is similar to that in the 7–9 years older Swedes; a strong contributor to the earlier diabetes onset as reported previously [30, 31].

The association of obesity with type 2 diabetes is a fact that has been known for decades, and along with obesity comes insulin resistance [32, 33, 34]. In this study, BMI is shown to have a great impact on insulin secretion as well as insulin action in both the Iraq born and the Swedish group.

The prevalence of type 2 diabetes and obesity is increasing worldwide. However, T2D is also increasing among non-obese Asians and some studies suggest that T2D among non-obese individuals is as high as 60–80% in some Asian countries, suggesting genetic inheritance of insulin resistance [35, 36, 37]. The factors predisposing non-obese Middle Eastern individuals to type 2 diabetes are not fully characterized and understood but several studies suggest an inherited defect in beta cell compensation prior to the onset of T2D [38, 39, 40, 41, 42]. Since this study has a cross-sectional design, we can only observe that the Iraqi-born individuals seem to have an earlier decline in insulin secretion. This is in agreement with earlier studies showing that type 2 diabetes in non-obese individuals has a stronger association with insulin secretion defects rather than insulin action defects [35].

Considering the strong heritability for insulin secretion in Middle Eastern immigrants [30], the pattern of insulin secretion among younger Middle Eastern subjects is of interest for future studies to explore if the decline is similar in adolescents, but appears at an earlier age in adults, or if the Middle Eastern population have an inborn lower insulin secretion from birth.

### 4.2 Strengths of the study

Health care is equally accessible for immigrant and native populations in Sweden, and this is one of the strengths of this study, as accessibility to health care cannot explain the differences in glucose metabolism observed in this study. The Matsuda indices show good reliability and correlation in several studies to OGTT, since Matsuda indices are calculated based on OGTT data, they reflect both hepatic and muscular insulin sensitivity.

#### 4.2.1 Limitations of the study

A limitation of the study is the potential selection biases, such as the participants’ health and social status, and it was seen that Iraqi men participated to a higher degree than Iraqi women. Another limitation is the cross-sectional design, where individuals cannot be followed over time, and age effects may be confounded by the effects of birth cohort.

#### 4.2.2 Strengths in relation to other studies

Several studies have been conducted indicating the higher prevalence of diabetes and other metabolic risk factors among immigrant populations in western countries. A deeper understanding of the mechanism is illustrated by the current study. We have tried to understand the pathophysiological mechanisms before diabetes onset contributing to the high type 2 diabetes risk in Middle Eastern immigrants. Compared to other studies, we do not only shed light on the traditional risk factors but also on the age profile of insulin action and secretion among this immigrant group.

### 4.3 Representativeness of the study sample

The age and sex distribution in participating immigrants from Iraq did not differ compared to the eligible background population. The Swedish-born participants were older (49.3 vs. 45.5 years) but the sex distribution did not differ from the eligible native Swedish population.

### 4.4 Meaning of the study: possible explanations and implications for clinicians and policymakers

Considering the high proportion of Middle Eastern immigrants who are at risk of developing type 2 diabetes, a great cost will be imposed on healthcare and society as a consequence of loss of productivity years and life-years. A proper understanding of contributing risk factors across ethnicities is required to tailor preventative actions and treatment of this population. For instance, being overweight is shown here to have a great effect on insulin secretion and action and, if avoided at younger ages, many healthy and productive life years could be gained.

### 4.5 Unanswered questions and future research

Longitudinal studies with detailed data on exposure and circumstances are needed in order to better understand the causality and dynamics of insulin secretion and action.

## 5 Conclusion

The study shows that there seem to be differences across ethnicities in regard to insulin secretion and action with Iraqi immigrants showing a decrease in the beta-cell function as well as lower insulin sensitivity as compared to Swedes. The level of insulin action in the youngest Iraqis was at the level of that of the oldest Swedes. However, over the age span considered, the changes in insulin secretion and insulin action appear similar regardless of ancestry.

## Declarations

### Author contribution statement

Louise Bennet: Conceived and designed the experiments; Performed the experiments; Analyzed and interpreted the data; Contributed reagents, materials, analysis tools or data; Wrote the paper.

Nadine Fadhel Dhaher: Analyzed and interpreted the data; Wrote the paper.

Anton Nilsson, Nael Shaat: Analyzed and interpreted the data; Contributed reagents, materials, analysis tools or data; Wrote the paper.

### Funding statement

Louise Bennet was supported by The Swedish Research Council (Linné grant) [LUDC 349-2006-237, Exodiab 2009-1039, 2019-00978].

Louise Bennet was supported by ALF Grants [20101641, 20101837, 162641].

Louise Bennet was supported by The Swedish foundation for Strategic Research [LUDC IRC15-0067].

### Data availability statement

Data will be made available on request.

### Declaration of interest's statement

The authors declare no conflict of interest.

### Additional information

No additional information is available for this paper.

## Appendix A Supplementary data

Supplement1 paper1 Supplement1 paper1
