# Supplementary material for: Insulin secretion and action with increasing age - A comparison between Middle Eastern immigrants and native Swedes
Source: Heliyon. 2022 Oct 3;8(10):e10913. doi: 10.1016/j.heliyon.2022.e10913 (PMC9563179; doi:10.1016/j.heliyon.2022.e10913)
Supplement: Supplement1 paper1 [file mmc1.docx]

Supplement 1

Population informed about the study by phone or mail:

Iraqis: n=2,924 (♂ 1798, ♀ 1126)

Swedes: n=2,372 (♂ 1244, ♀ 1128)

Insulin Sensitivity index:

Iraqis. n=1,212 (♂ 706, ♀ 506)

Swedes: n=704 (♂ 372, ♀ 332)

Oral Disposition index:

Iraqis. n=1,193 (♂ 696, ♀ 497)

Swedes: n=688 (♂ 367, ♀ 321)

Diabetes diagnosis based on medication, HbA1c, fasting blood sugar or OGTT result:

Iraqis: n=173 (♂ 111, ♀ 62)

Swedes: n=45 (♂ 29, ♀ 16)

Drop-outs:

Iraqis: n=465 (♂ 269, ♀ 196)

Swedes: n=197 (♂ 110, ♀ 87)

Not meeting the inclusion criteria:

Iraqis: n=30 (♂ 16, ♀ 14)

Swedes: n=8 (♂ 4, ♀ 4)

Fasting samples and oral glucose tolerance tests:

Iraqis: n=1,344 (♂ 779, ♀ 565)

Swedes: n=757 (♂ 400, ♀ 357)

Physical examination, blood samples and questionnaires:

Iraqis: n=1,398 (♂ 819, ♀ 579)

Swedes: n=757 (♂ 400, ♀ 357)

Agreed to participate:

Iraqis: n=1,863 (♂ 1088, ♀ 775)

Swedes: n=954 (♂ 488, ♀ 466)

Eligible study population:

Iraqis: n=2,894 (♂ 1782, ♀ 1112)

Swedes: n=2,364 (♂ 1240, ♀ 1124)

Not meeting criteria glc-30 > f-glc and glc-30 > 4.44

Iraqis: n=132, 151 (♂ 73, ♀ 59 for ISI), (♂ 83, ♀ 68 for DIo)

Swedes: n=53, 69 (♂ 28, ♀ 25 for ISI), (♂ 33, ♀ 36 for DIo)

A flowchart describing the recruitment of MEDIM participants and response rate.
